# Evaluation of lime and hydrothermal pretreatments for efficient enzymatic hydrolysis of raw sugarcane bagasse

**DOI:** 10.1186/s13068-015-0384-y

**Published:** 2015-12-02

**Authors:** Maira Prearo Grimaldi, Marina Paganini Marques, Cecília Laluce, Eduardo Maffud Cilli, Sandra Regina Pombeiro Sponchiado

**Affiliations:** Department of Biochemistry and Technology Chemistry, Institute of Chemistry, São Paulo State University-UNESP, R. Prof. Francisco Degni 55, Araraquara, SP CEP 14800–060 Brazil

**Keywords:** Sugarcane bagasse, Lime pretreatment, Hydrothermal pretreatment, Chemical composition, Scanning electron microscopy, X-Ray diffraction, Thermogravimetric analysis, Enzymatic hydrolysis

## Abstract

**Background:**

Ethanol production from sugarcane bagasse requires a pretreatment step to disrupt the cellulose-hemicellulose-lignin complex and to increase biomass digestibility, thus allowing the obtaining of high yields of fermentable sugars for the subsequent fermentation. Hydrothermal and lime pretreatments have emerged as effective methods in preparing the lignocellulosic biomass for bioconversion. These pretreatments are advantageous because they can be performed under mild temperature and pressure conditions, resulting in less sugar degradation compared with other pretreatments, and also are cost-effective and environmentally sustainable. In this study, we evaluated the effect of these pretreatments on the efficiency of enzymatic hydrolysis of raw sugarcane bagasse obtained directly from mill without prior screening. In addition, we evaluated the structure and composition modifications of this bagasse after lime and hydrothermal pretreatments.

**Results:**

The highest cellulose hydrolysis rate (70 % digestion) was obtained for raw sugarcane bagasse pretreated with lime [0.1 g Ca(OH)_2_/g raw] for 60 min at 120 °C compared with hydrothermally pretreated bagasse (21 % digestion) under the same time and temperature conditions. Chemical composition analyses showed that the lime pretreatment of bagasse promoted high solubilization of lignin (30 %) and hemicellulose (5 %) accompanied by a cellulose accumulation (11 %). Analysis of pretreated bagasse structure revealed that lime pretreatment caused considerable damage to the bagasse fibers, including rupture of the cell wall, exposing the cellulose-rich areas to enzymatic action.

**Conclusion:**

We showed that lime pretreatment is effective in improving enzymatic digestibility of raw sugarcane bagasse, even at low lime loading and over a short pretreatment period. It was also demonstrated that this pretreatment caused alterations in the structure and composition of raw bagasse, which had a pronounced effect on the enzymes accessibility to the substrate, resulting in an increase of cellulose hydrolysis rate. These results indicate that the use of raw sugarcane bagasse (without prior screening) pretreated with lime (cheaper and environmentally friendly reagent) may represent a cost reduction in the cellulosic ethanol production.

## Background

Following the world trend for more research on alternative fuels, Brazilian sugar, and ethanol industry has shown interest in sustainable technologies that can be aggregated to its productive chain. Efforts are currently being directed toward the inclusion of sugarcane bagasse, straw, and tops in the production cycle of second-generation ethanol [1–6]. Due to its abundance and low cost, sugarcane bagasse is considered an interesting raw material for bioconversion since the sugars contained in cellulose and hemicellulose fractions represent the substrates that can be used by yeast for cellulosic ethanol production. The use of this biomass would bring economic and ecological benefits because it allows increasing production without the need to increase the planted area and solving the problem of disposal of this residue [4, 7–9]. Due to its recalcitrant structure, pretreatment is a necessary step to change some structural characteristics of bagasse and to increase cellulose accessibility to hydrolytic enzymes in order to provide high yields of fermentable sugars for subsequent fermentation [10–16].

Considering that pretreatment represents the second most expensive step in the conversion of biomass into ethanol, the great challenge of this technology is to find an appropriate strategy to disrupt the lignocellulosic complex, allowing enzymatic hydrolysis with low loads of enzymes and low conversion times, in a cost-effective manner and environmentally sustainable [5, 14, 17]. Physical, chemical, physicochemical, and biological pretreatments are currently applied to different lignocellulosic biomass but the choice of appropriate pretreatment must take into account some factors, such as (1) increase in accessible surface area, (2) cellulose decrystallization, (3) modification of the lignin structure, (4) solubilization of hemicellulose and/or lignin, (5) no significant hemicellulose and cellulose degradation; (6) increased yield of fermentable sugars after enzymatic hydrolysis; (7) low generation of toxic compounds potentially inhibitory for yeasts; (8) reduction of biomass size is not required; (9) use of cheaper and environmentally friendly reagents; and (10) catalyst recovery and/or solvent recycling. These factors significantly affect costs associated with the pretreatment step [3, 8, 11, 12, 18, 19].

Hydrothermal and alkaline pretreatments have emerged as effective methods in preparing the lignocellulosic biomass for enzymatic hydrolysis because they operate under mild temperature and pressure conditions, have less sugar degradation compared with acid pretreatment and also cause delignification and deacetylation depending on the pretreatment severity, greatly enhancing carbohydrate digestibility [10, 17]. Hydrothermal pretreatment, also called liquid hot water pretreatment, has economic advantages and is environmental friendly because it uses only water as reaction medium without additional chemicals, does not require special non-corrosive reactor or preliminary feedstock size reduction and produces small amounts of undesired degrading compounds, such as furfural. The main effect of this pretreatment is to solubilize mainly hemicellulose and to cause structural changes in lignin, which contribute to the reduction of biomass recalcitrance, making cellulose more susceptible to enzymatic action [9, 12, 15, 20]. Laser et al. [21] reported that liquid hot water pretreatment promoted 86 % cellulose conversion by simultaneous saccharification and fermentation, 82 % xylan recovery from sugarcane bagasse and no inhibition of the glucose fermentation rate. Lime pretreatment is another attractive method because calcium hydroxide is much cheaper than other alkalis, has low toxicity to the environment and can be easily recovered from hydrolysate as insoluble calcium carbonate with carbon dioxide and subsequently, calcium hydroxide can be regenerated using lime kiln technology. This pretreatment is very effective in the removal of amorphous substances, such as lignin and hemicellulose, because it cleaves α- and β-ether bonds in phenolic units and β-ether linkages in non-phenolic units, which causes disruption of the lignin structure and changes in the degree of polymerization and crystallinity of cellulose, enhancing enzymes accessibility to the substrate. Compared with acid and hydrothermal pretreatments, alkaline methods cause less degradation of cellulosic fraction, which results in greater release of sugars during enzymatic hydrolysis [10, 12, 16, 18]. Rabelo et al. [22] reported that higher yields of total reducing sugars were obtained after enzymatic hydrolysis of lime-pretreated sugarcane bagasse compared to that treated with alkaline peroxide hydrogen.

The effectiveness of pretreatment to increase the digestibility of lignocellulosic biomass is dependent on substrate structure and composition well as on pretreatments conditions. In this sense, the aim of this study was to evaluate the effect of the hydrothermal and lime pretreatments on structure, composition, and susceptibility to enzymatic hydrolysis on raw sugarcane bagasse coming from mill without prior screening. Although hydrothermal and lime pretreatments have been studied on different types of lignocellulosic biomass, only one study was performed using sugarcane bagasse as it comes from mill [23]. The use of such bagasse may contribute to reduce operating costs because it is not submitted to any preparation step (such as screening), which are expensive and time consuming. In this paper, we show that lime pretreatment was more effective than hydrothermal pretreatment to promote higher cellulose digestibility rates. It was also demonstrated that this increased hydrolysis rate is related to changes in the structure and composition of bagasse occurred during lime pretreatment.

## Results and discussion

### Enzymatic hydrolysis of pretreated sugarcane bagasse

To evaluate the efficiency of the hydrothermal and lime pretreatments to enhance the digestibility of raw sugarcane bagasse, the rates of conversion of cellulose into glucose during enzymatic hydrolysis of pretreated bagasse were measured.

**Fig. 1:**
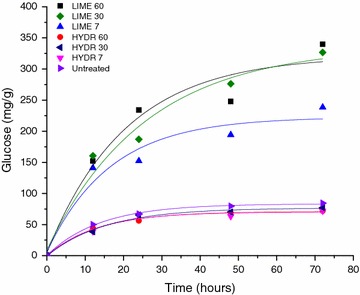
Glucose release during the enzymatic hydrolysis of raw sugarcane bagasse submitted to lime (LIME) and hydrothermal (HYDR) pretreatments for 7, 30, and 60 min compared to untreated bagasse. The *lines* represent an exponential fit using equation: *y* = *y*_0_ + *A* × exp(*R*_0_ *x*)

As shown in Fig. 1, the highest hydrolysis rates were obtained for bagasse pretreated with lime and the pretreatment time exerted more influence on glucose release from bagasse pretreated with lime compared to hydrothermal pretreatment and untreated bagasse. After 72 h of enzymatic hydrolysis, glucose released from bagasse pretreated with lime reached 256; 320; and 384 mg/g dry bagasse, corresponding to 52, 57, and 70 % cellulose digestion at 7, 30, and 60 min of pretreatment, respectively. For hydrothermal pretreatment, values were 83; 97 and 101 mg/g dry bagasse, whose cellulose digestion percentage varied from 17 to 21 %, considering the same pretreatment time. These values were very similar to those obtained for hydrolysis of untreated bagasse.

Our results also showed evidence that cellulose digestion depends on the pretreatment time. Sugarcane bagasse submitted to lime pretreatment exhibited an increase of 208, 223, and 280 % in glucose release compared to hydrothermal pretreatment after 7, 30, and 60 min of pretreatment, respectively. The statistical analysis of data confirmed that the variables studied (time and pretreatment) and interaction between them have a significant effect (*p* < 0.05) on cellulose digestion. Time is very important parameter for an economic analysis of the process because it allows evaluating if the increase in glucose released during saccharification compensates the energy cost when using longer pretreatment periods [4, 5, 19]. Studies conducted by Playne [32] obtained 60 % cellulose digestion when sugarcane bagasse was pretreated with 0.12 Ca(OH)_2_/g dry raw during 8 days at 20 °C. Fuentes et al. [33] and Rabelo et al. [34] obtained glucose yield of 228.45 mg/g dry raw for sugarcane bagasse pretreated with 0.4 g Ca(OH)_2_/g dry biomass for 90 h at 90 °C. Chang, Holtzapple, and Nagwany [24], using sugarcane bagasse pretreated with 0.1 gCa(OH)_2_/g dry raw, obtained yield of 300 mg/g dry bagasse after 1 h of pretreatment at 120 °C. In the present study, higher glucose yield (320 mg/g dry raw) was obtained when bagasse was treated with the same amount of lime (0.1 g Ca(OH)_2_/g dry raw) but within a shorter time (30 min).

### Chemical composition of pretreated sugarcane bagasse

It is known that the rate of enzymatic hydrolysis of lignocellulosic substrates is related to changes in biomass composition and structure occurred during pretreatments. In order to explain the different percentages of cellulose digestion obtained, the cellulose, hemicellulose, and lignin contents of the bagasse before and after lime and hydrothermal pretreatments were determined. As can be seen in Table 1, the raw sugarcane bagasse (control) used in this study presented cellulose (45 %), hemicelluloses (33 %), and lignin (24 %) composition similar to those reported in literature for the same material, whose values vary from 39 to 45 % for cellulose, 26–36 % for hemicellulose and 11–25 % for lignin [35].

**Table 1:** Chemical composition of the raw sugarcane bagasse after hydrothermal (HYDR) and lime (LIME) pretreatments

Pretreatment	Lignin (%)	Hemicellulose (%)	Cellulose (%)	Pretreatment yield^a^ (%)
Control^b^	23.77 ± 0.30	32.77 ± 2.25	44.49 ± 1.33	100
LIME 7	19.02 ± 0.28	35.33 ± 0.60	44.98 ± 0.45	75.0 ± 1.4
LIME 30	19.18 ± 1.95	31.23 ± 1.17	49.55 ± 1.27	68.2 ± 3.2
LIME 60	16.70 ± 0.52	31.86 ± 0.25	49.56 ± 0.12	51.1 ± 1.6
HYDR 7	24.28 ± 0.26	33.08 ± 0.48	44.26 ± 1.63	89.0 ± 1.4
HYDR 30	23.96 ± 0.68	31.75 ± 0.12	43.81 ± 0.33	85.2 ± 1.6
HYDR 60	23.74 ± 0.33	30.25 ± 1.13	43.11 ± 0.97	70.5 ± 0.0

Contents of the each fraction are expressed an average ± standard deviation of two replicates

^a^The pretreatment yield refers to the insoluble solids remaining after pretreatment as a percentage of the initial material

^b^Control = raw sugarcane bagasse untreated

Analyzing the composition of bagasse submitted to different pretreatments, it was observed that the greatest changes in lignin, hemicellulose, and cellulose contents occurred in bagasse pretreated with lime compared to hydrothermally pretreated bagasse. After 60 min of lime pretreatment, the lignin percentage decreased from 23.77 to 16.7 %, the hemicelluloses content remained practically unchanged (varying from 32.77 to 31.86 %), while cellulose content increased from 44.49 to 49.56 % (Table 1).

These alterations resulted in greater mass reduction in bagasse pretreated with lime compared to hydrothermally pretreated bagasse. The pretreatment yield, expressed as percentage of initial material, ranged from 51 to 75 % for lime pretreatment and from 70 to 89 % for hydrothermal pretreatment, being proportional to the increase in pretreatment time (Table 1). Rabelo, Maciel, and Costa [23] also evaluated the effect of lime pretreatment on sugarcane bagasse and obtained pretreatment yield of 58.73 % using 0.4 g Ca(OH)_2_/g dry bagasse for 90 h at 90 °C. We obtained lower yield (51.1 %) with low lime loading 0.1 g Ca(OH)_2_/g dry bagasse and also shorter pretreatment time (60 min).

**Fig. 2:**
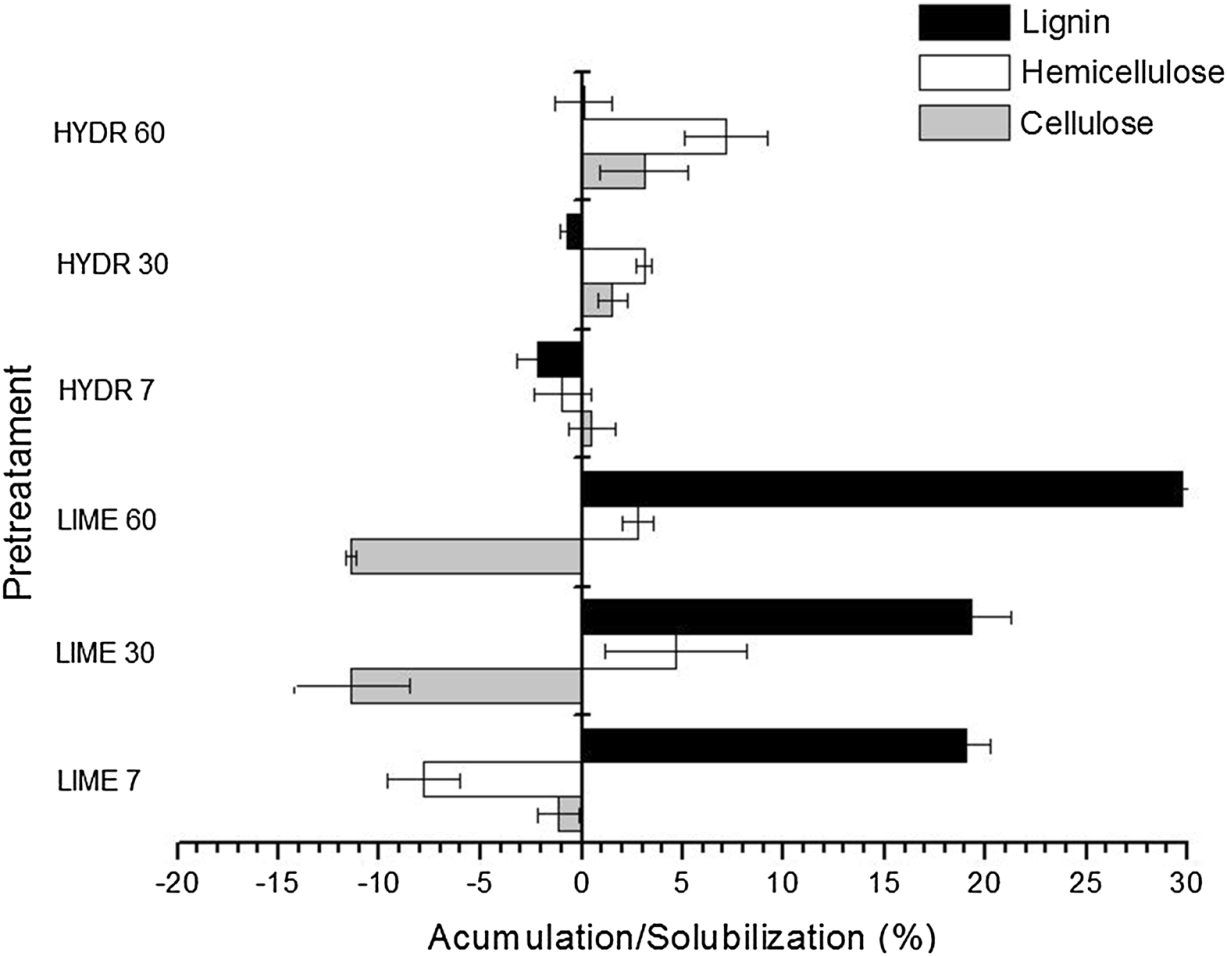
Accumulation and solubilization of lignin, hemicellulose, and cellulose of raw sugarcane bagasse submitted to hydrothermal and lime pretreatments

Figure 2 shows more clearly that lime pretreatment affected mainly the lignin fraction, which was gradually removed with increasing pretreatment time, reaching 30 % of solubilization after 60 min, while no lignin solubilization occurred in hydrothermal treatment. The solubilization of the hemicellulose fraction was lower for both pretreatments, ranging from 3 to 7 % after 30 and 60 min of pretreatment. It was also observed that hydrothermal pretreatment removed a small portion of cellulose, and only 1.5 to 3 % was solubilized. In contrast, the cellulose content in bagasse pretreated with lime increased to 11 % after 30 and 60 min of pretreatment.

Our results corroborate those of other studies, such as Chang et al. [36, 37], Mosier et al. [38], Hendriks and Zeeman [10], Rabelo et al. [23]. They also reported that lime pretreatment has major effect on delignification, accompanied by a small dissolution of hemicelluloses, but cellulose in not affected in this pretreatment. The lack of cellulose degradation can be explained by its high polymerization and crystallinity degree and low reactivity with alkali due to its relative stability under alkaline conditions. However, hemicellulose is more labile and consequently dissolution of this polysaccharide can occur [11, 39–42].

Cellulose enrichment has a great importance for the production of ethanol from biomass, because no degradation of the cellulosic fraction results in higher concentration of fermentable sugars after enzymatic hydrolysis of cellulose, which is essential for economic viability of the bioconversion process [3, 5, 9, 43]. Similar study was carried out by Chang, Nagwani, and Holtzapple [24] using sugarcane bagasse pretreated with 0.1 g Ca(OH)_2_/g dry biomass at 120 °C for 1 h, achieving 19 % of lignin solubilization, 1 % of hemicellulose solubilization, and 7 % of cellulose accumulation. In the present work, the same pretreatment conditions described by these authors were used, but our results were superior using raw sugarcane bagasse: 30 % of lignin and 5 % of hemicellulose were removed, resulting in 11 % increase in the cellulose content. This variation in results may be related to difference in particle size, processing conditions and sugarcane cultivars [3, 44].

In order to assess the significance of the effects of pretreatment time and type on the solubilization of lignin, hemicellulose, and cellulose fractions in pretreated bagasse, analysis of variance (ANOVA) was performed using 95 % confidence level. The analysis of data showed that the variables studied (time and pretreatment) as well as interaction between them exerted significant influence on the delignification of pretreated bagasse, with *p* value less than 0.05. The same behavior was observed for cellulose, all the variables studied and their interactions are significant (*p* < 0.05), and the effect of treatment, followed by the treatment/time interaction caused greater change in this fraction. For hemicellulose, only time was significant (*p* < 0.05) and the treatment/time interaction did not significantly influence the solubilization of this fraction (*p* > 0.05). Thus, this analysis confirmed that the lime pretreatment affected the lignin and cellulose fractions, inducing high lignin solubilization and cellulose accumulation proportional to the pretreatment time.

Several studies have shown that variations in the composition of biomass submitted to different pretreatments can be related to pH variations and holding time conditions, which affect the pretreatment severity and consequently have a great effect on enzymatic hydrolysis [18, 45, 46]. In present study, the “severity factor” was used as parameter to compare the effects of lime and hydrothermal pretreatments on raw sugarcane bagasse.

**Fig. 3:**
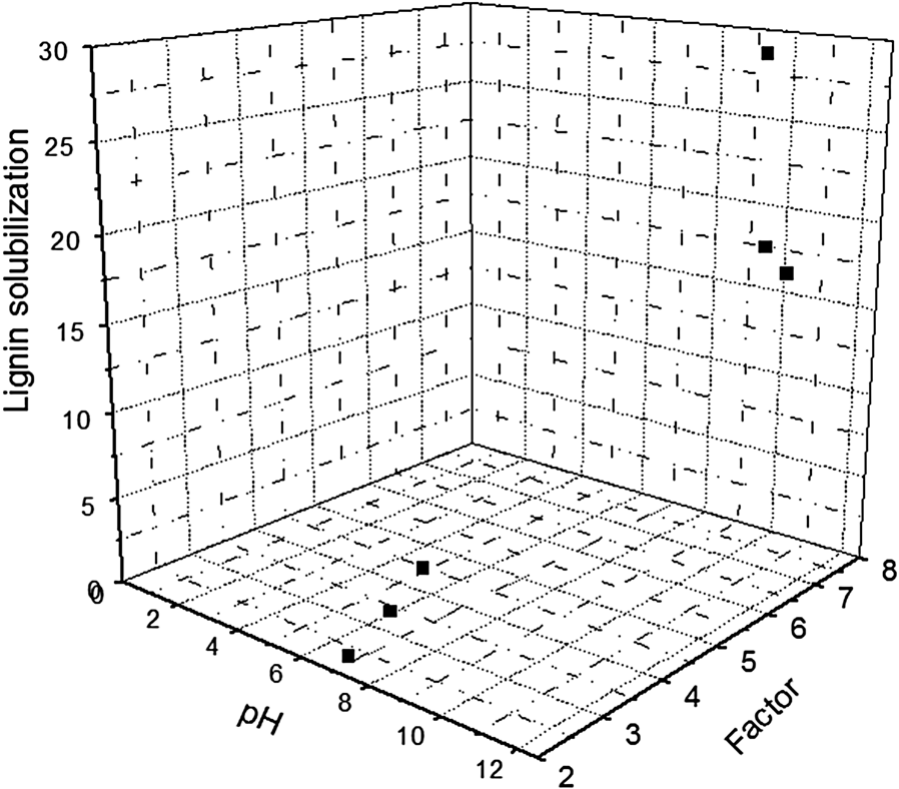
Lignin solubilization as responses to pH and calculated severity factor in different times of pretreatments

Figure 3 shows lignin solubilization as responses to severity factor calculated and pH obtained in hydrothermal (pH 2.9, 3.6, and 4.4) and lime (pH 6.2, 6.5, and 6.7) pretreatments for times of 7, 30 and 60 min, respectively.

As can be seen in Fig. 3, lime pretreatment exhibited higher severity factor (6.2–6.5) compared to hydrothermal pretreatment (2.9 e 4.4). It was also observed that higher severity factor is related to increased lignin solubilization and alkaline pH, resulting from lime pretreatment. These results are consistent with other studies that consider pretreatment pH as an important factor when analyzing the pretreatment severity on lignin solubilization. When pretreatment is carried out at alkaline pH under mild conditions (below 140 °C), it affect the biomass composition, reducing mainly lignin content due to cleavage of ester linkages joining phenolic acids: the nucleophilic acyl substitution of ester bonds normally takes place during reaction with an alkaline salt (calcium hydroxide). This promotes lignin solubilization, thereby making biomass more digestible, resulting in an increased hydrolysis yield of glucose as consequence of the high enzyme catalyzed cellulose degradation [18, 47].

**Fig. 4:**
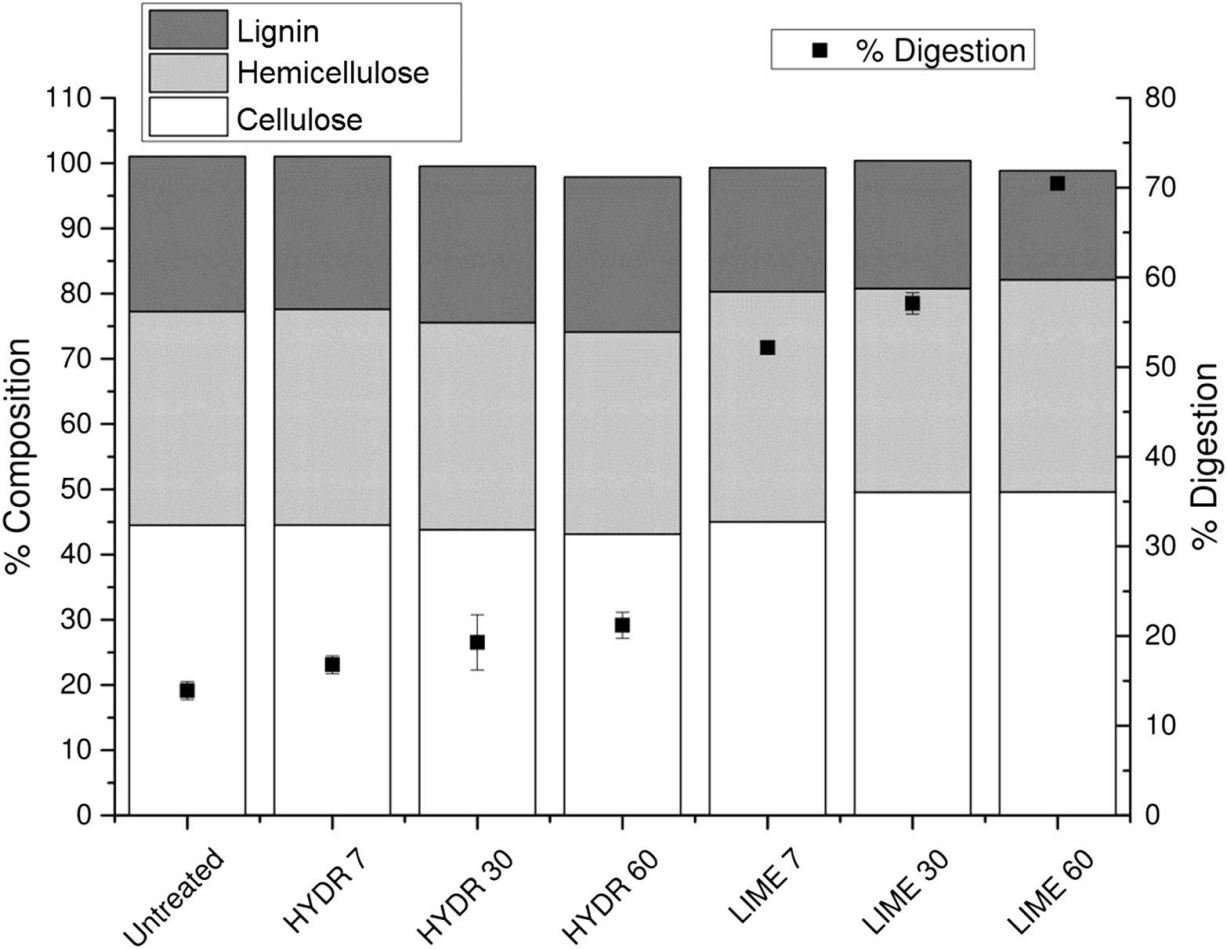
Chemical composition and percentage of cellulose digestion of raw sugarcane bagasse submitted to hydrothermal (HYDR) and lime (LIME) pretreatments

Our results confirmed that lime pretreatment had a more pronounced effect on the efficiency of enzymatic hydrolysis of raw bagasse compared to hydrothermal pretreatment (Fig. 1). The high percentage of cellulose digestion obtained in bagasse pretreated with lime indicates that the cellulosic fraction is more accessible to enzymes probably due to alterations in bagasse composition after pretreatment. As can be seen in Fig. 4, the pretreatment with lime promoted greater bagasse delignification (30 % of solubilization after 60 min), resulting in higher cellulose digestion. Bagasse pretreated with lime reached 70 % of cellulose digestion, while in hydrothermal pretreated bagasse only 21 % of cellulose was converted into glucose after 60 min of pretreatment. This value was very close to that obtained for untreated bagasse (14 % of cellulose digestion).

According to literature, the presence of lignin in biomass restricts enzymatic hydrolysis because it acts as a physical barrier preventing the accessibility of cellulase to substrate and also as a competitive adsorbent for cellulases, reducing the activity of adsorbed enzymes [15, 43]. Chang and Holtzapple [48] reported that there are correlations between enzymatic digestibility and three structural factors of biomass: lignin content, crystallinity, and acetyl content. They concluded that (1) extensive delignification is sufficient to obtain high digestibility regardless of acetyl content and crystallinity, (2) delignification and deacetylation remove parallel barriers to enzymatic hydrolysis, and (3) crystallinity significantly affects initial hydrolysis rates but has less effect on sugar yields. Lee and Fan [49] reported that the enzymatic hydrolysis rate depends on enzyme adsorption and the effectiveness of adsorbed enzymes, instead of the diffusive mass transfer of enzymes. Lignin removal improves enzyme effectiveness by eliminating nonproductive adsorption sites, increasing access to cellulose and hemicellulose. In addition, alkaline saponification of acetyl and uronic ester groups in hemicellulose reduces the steric hindrance of hydrolytic enzymes and also contributes to enhance the enzymatic accessibility of polysaccharides [11, 42]. Thus, our results confirmed that the high glucose yields obtained after enzymatic hydrolysis of raw bagasse pretreated with lime is probably related to the low lignin and hemicellulose contents and the high cellulose content of bagasse after pretreatment.

### Structural analysis in the pretreated sugarcane bagasse

**Fig. 5:**
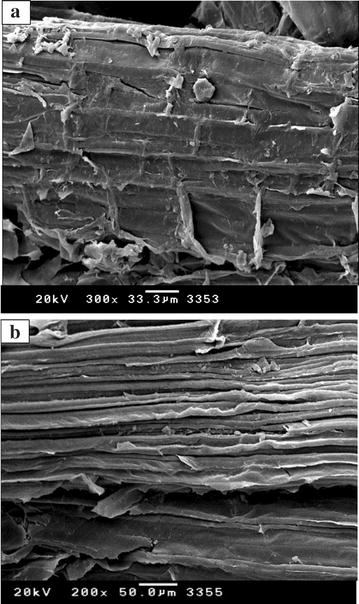
Scanning electron microscopy of raw sugarcane bagasse without pretreatment (**a**) and pretreated with lime (**b**)

Several studies have shown that lime pretreatments had a remarkable effect on lignocellulosic biomass structure [16, 23, 50]. Calcium ions extensively crosslinked lignin molecules under alkaline conditions, disrupting of chemical bonds stiffening lignocellulose by removing lignin and acetyl groups from hemicelluloses, which results in increased biomass porosity, effectively improving the enzymatic digestibility of pretreated material [48, 51, 52]. In the present study, modifications on the surface of bagasse pretreated with lime for 30 and 60 min were analyzed by scanning electron microscopy (Fig. 5).

From the analysis of Fig. 5, it was observed that, although tissue integrity was maintained to some extent, there are signs of fragmentation on the surface of bagasse pretreated with lime. For untreated samples, an ordered structure of matrix with whole cells was observed (Fig. 5a), while bagasse pretreated with lime presented considerable damage in its structure, including rupture of the cell wall, where inner parts of the cell were exposed (Fig. 5b). Disaggregation of cell bundles and the formation of long cellular structures in pretreated bagasse was also observed (Fig. 5b).

Rezende et al. [50], using a two-step pretreatment (diluted acid followed by alkaline treatment with NaOH) reported that in bagasse submitted to NaOH at concentrations lower than 0.5 %, the cell bundles start to dismantle and fibers become detached from one other. When NaOH concentrations above 0.5 % are used, the unidirectional separation of the cell wall bundles on the pretreated samples was observed. These results showed that lignin removal caused destructuring of the bagasse cell wall, which occurs in two levels. The first level refers to the loss of cohesion between neighboring cell walls, while the second level corresponds to degradation inside the cell wall, caused by peeling off and formation of holes. The results obtained in the present work are in agreement with these observations, since the disruption of fibers occurred to bagasse pretreated with lime (Fig. 5) was probably due to the removal of lignin after lime pretreatment (30 % delignification after 60 min), which resulted in increased conversion rate of cellulose into glucose (70 % saccharification), as shown in Fig. 4. Rezende et al. [50], also reported that these morphological alterations are important for improving cellulose hydrolysis because enzymatic action is hindered when bagasse fibers are packed and their surfaces are protected by lignin, which acts as an ‘enzymatic trap’, causing an unproductive adsorption of cellulases to the substrate.

Thermogravimetric analysis (TGA) was performed to better understand the effects of lime pretreatment on the structure of raw sugarcane bagasse. This analysis provides a useful tool to characterize bagasse fibers after pretreatment because the thermal behavior of lignocellulosic biomass is closely related to the chemical composition of fibers and physical characteristics of lignin, hemicellulose, and cellulose during thermal decomposition of pretreated bagasse [53].

**Fig. 6:**
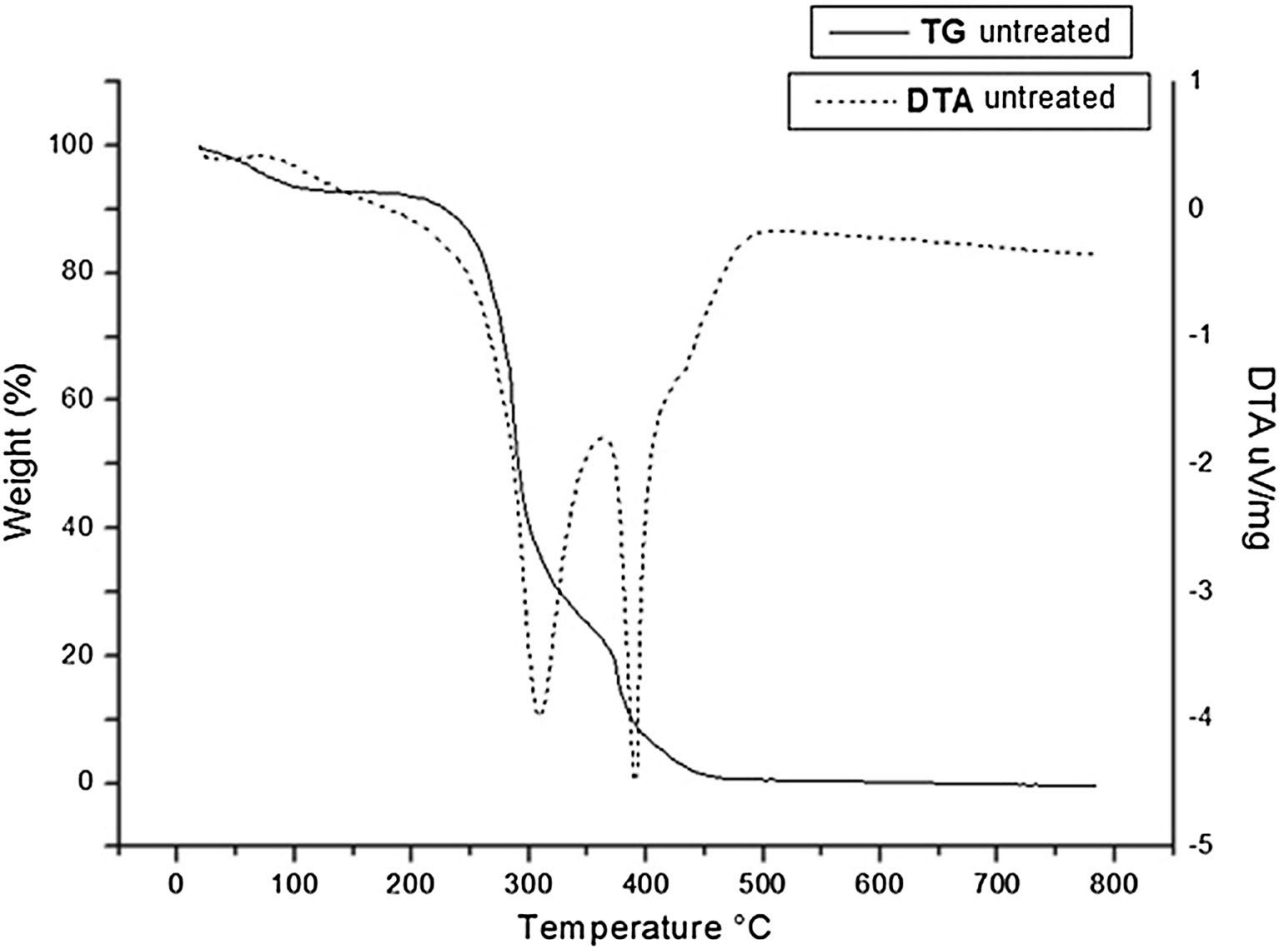
Thermal decomposition curve of untreated raw sugarcane bagasse. Conditions: 10 °C/min in air atmosphere alumina crucible (↓ exothermic peak)

**Fig. 7:**
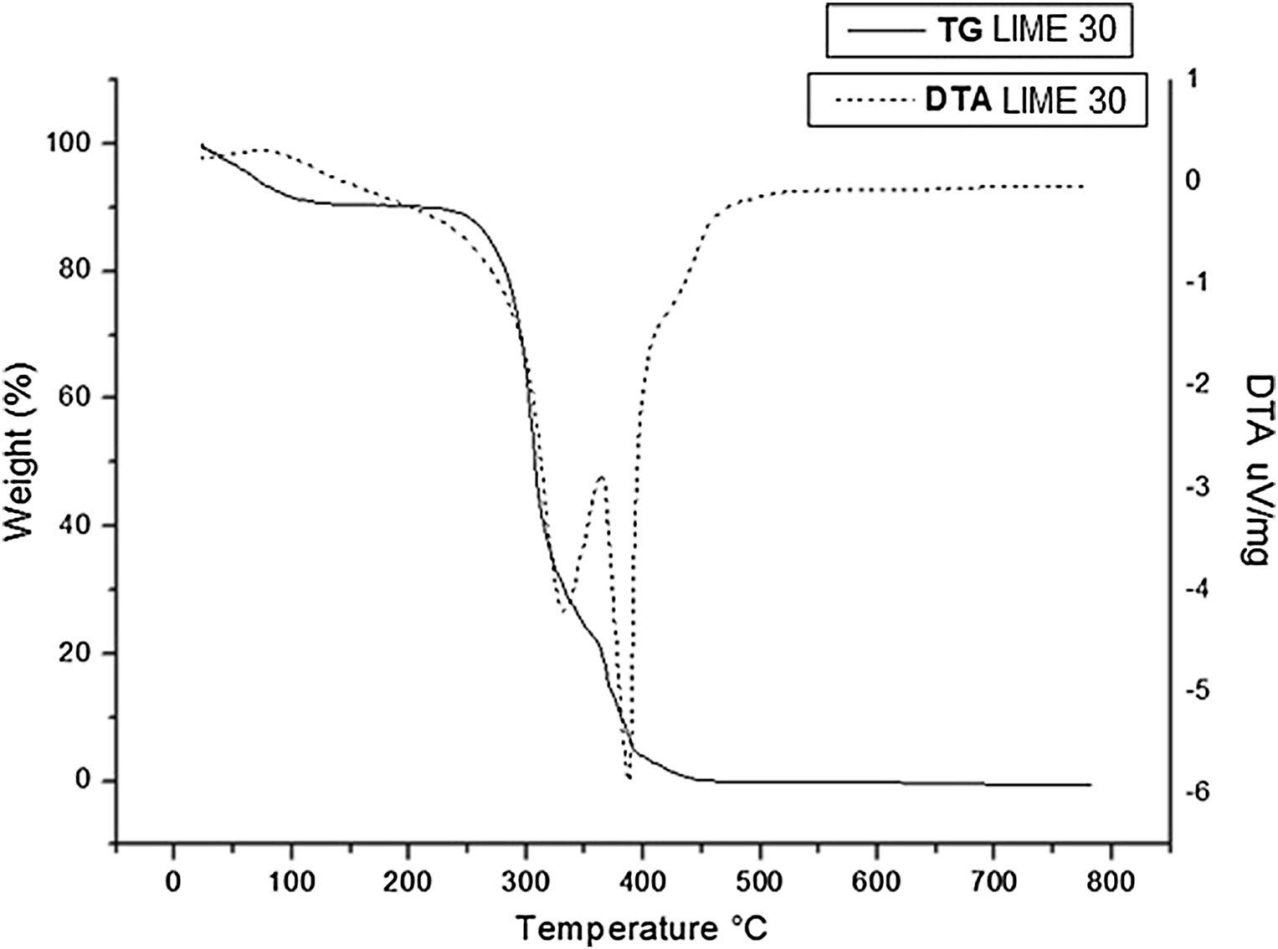
Thermal decomposition curve of raw sugarcane bagasse pretreated with lime for 30 min. Conditions: 10 °C/min in air atmosphere alumina crucible (↓ exothermic peak)

**Fig. 8:**
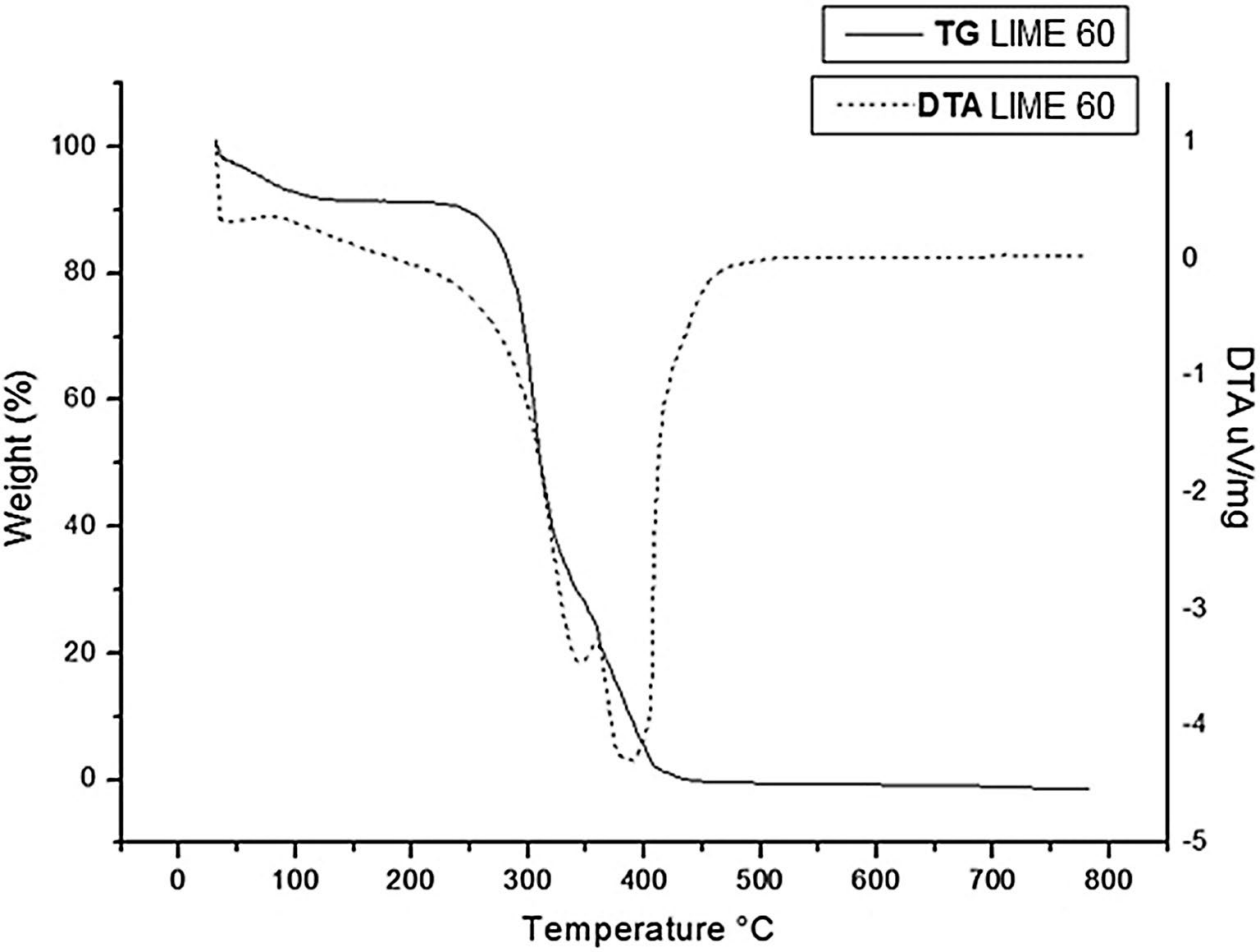
Thermal decomposition curve of raw sugarcane bagasse pretreated with lime for 60 min. Conditions: 10 °C/min in air atmosphere alumina crucible (↓ exothermic peak)

The Figs. 6, 7, and 8 show the thermogravimetric profiles of untreated bagasse and those pretreated with lime for 30 and 60 min. The DTA (Differential Thermal Analysis) curves showed three exothermic events, in agreement with the TG (Thermogravimetric) curves, indicating three weight loss stages: the first stage at 100 °C is attributed to the elimination of moisture accompanied by 8 % mass loss; the second stage occurs between 300 and 350 °C with weight loss of about 63–67 % and the third stage occurs at temperature range of 380–400 °C with weight loss from 22 to 27.4 %. The second and third stages are attributed to lignin, hemicellulose, and cellulose decomposition, which have similar stabilities. According to literature, hemicellulose decomposes first, followed by lignin and cellulose, and there is not a certain region for the event of breakdown of these fractions [54, 55].

As shown by the thermogravimetric analysis (TG/DTA), the distance between peaks related to the second and the third stage is smaller for pretreated samples than for untreated bagasse. These results suggest that lime pretreatment might have caused the decomposition of some components (cellulose, hemicellulose and lignin) of pretreated bagasse. This is in agreement with data obtained from compositional analysis of pretreated bagasse (Fig. 2), which showed that lime pretreatment promoted high delignification (30 % of solubilization) and also small hemicellulose degradation (5 % of solubilization) in pretreated bagasse.

In this study, X-ray diffraction analyses were performed to evaluate the impact of cellulose crystallinity on the digestibility of lime-pretreated bagasse. Cellulose crystallinity has been considered a biomass recalcitrance feature that, along with specific surface area, polymerization degree, cellulose sheathing by hemicelluloses, lignin, and acetyl contents, affect enzymatic hydrolysis performance in pretreated bagasse [11]. Crystallinity is strongly influenced by biomass composition as a consequence of the relative amounts of lignin, hemicellulose, and cellulose, which vary according to the pretreatment applied to the biomass [15].

**Fig. 9:**
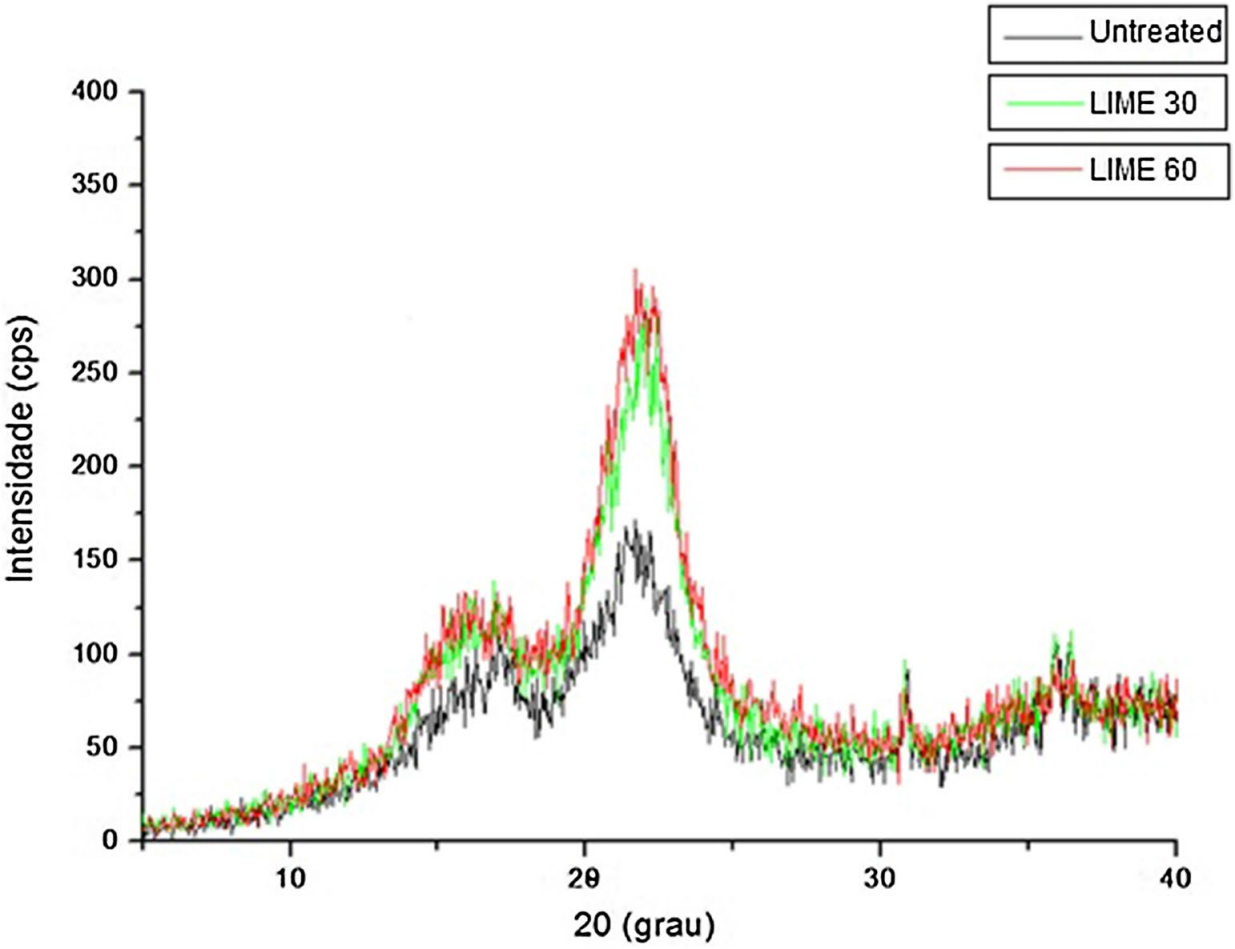
X-Ray diffraction analysis of raw sugarcane bagasse pretreated with lime for 30 min (LIME 30) and 60 min (LIME 60)

Figure 9 shows two peaks, one at 16° and another at 22° with full width at half maximum (FWHM). For bagasse pretreated with lime for 60 and 30 min, intensity of 300 and 285 cps with 61 % and 60 % of crystallinity, respectively, were obtained, while for untreated bagasse, peak intensity was 175cps with 43 % of crystallinity. These data indicate that lime pretreatment promoted an increase in the cellulose crystallinity degree (*I*_c_) of pretreated bagasse. Similar results were obtained by Chundawat et al. [56], who compared the effect of several pretreatments in the digestibility of lignocellulosic biomass. The authors observed that diluted acid, hydrothermal, steam explosion, and lime pretreatments generally result in relative increase of cellulose crystallinity compared to untreated control. According to Ishizawa et al. [57]. and Sheikh et al. [58], the increase in cellulose crystallinity was caused by the lignin removal, which exposed the crystalline cellulose core and increased the glucan content in the solid fraction of pretreated biomass. Other studies have also reported that the crystallinity degree increased slightly when amorphous components (such as lignin and hemicelluloses) were removed [12, 48, 59]. Then the increase in cellulose crystallinity obtained in the present study may be a consequence of the high delignification percentage (30 % of lignin solubilization) of bagasse pretreated with lime (Fig. 2).

Taking into account all results obtained in this present study, it could be inferred that they are consistent with the model proposed by Chang and Holtzapple [48]. According to this model, enzymes flow through pipes before reaching the substrate tank and the flow through each pipe is regulated by a large valve (lignin content). When the lignin valve is opened (i.e., most lignin is removed), enzymes can easily flow through the wide pipe and arrive at the substrate tank to be adsorbed on the substrate surface. In contrast, if the lignin valve is closed (i.e., none or little lignin is removed), enzymes can hardly flow through the wide pipe. After enzymes arrive at the substrate tank, they begin to work. How fast they work (i.e., enzyme effectiveness) depends on the substrate crystallinity. If the substrate is amorphous, enzyme effectiveness is high and enzymes are adsorbed on the substrate more rapidly. In contrast, if the substrate is highly crystalline, enzyme effectiveness is low and enzymes work slowly. In this model, the extent of enzymatic hydrolysis depends on two factors: how many enzymes arrive at the substrate tank and how fast they work. In the present study, our results showed that lime pretreatment promoted greater reduction in lignin content of raw sugarcane bagasse, allowing enough enzymes to reach carbohydrate polymers (cellulose), although they are not as effective due to the high substrate crystallinity, the amount of enzymes adsorbed on the substrate was sufficient to achieve high cellulose conversion (70 % digestion) after a 3-day period.

## Conclusion

From the results obtained in this study, it could be conclude that lime pretreatment was more efficient to promote greater digestibility rates of raw sugarcane bagasse (70 % cellulose digestion) compared with hydrothermal pretreatment (21 % cellulose digestion). This increase in the cellulose hydrolysis rate was mainly due to lignin and hemicellulose removal (30 and 5 % solubilization, respectively) and the increased cellulose content (11 % enrichment) of bagasse pretreated with lime. Analysis of pretreated bagasse structure revealed that lime pretreatment caused considerable damage in bagasse fibers, including rupture of the cell wall, exposing cellulose-rich areas to enzymatic action and consequently contributing to the high conversion rate. Comparing with literature, our results showed that it is possible to obtain high yield of fermentable sugars (384 mg glucose/g dry bagasse) using raw sugarcane bagasse pretreated with low lime loading (0.1 g Ca(OH)_2_/g dry bagasse) and shorter pretreatment time (60 min) at 120 °C. These results have a substantial importance for the production of cellulosic ethanol because the use of raw sugarcane bagasse (without prior screening) pretreated with lime (cheaper and environmentally friendly reagent) may represent a cost reduction in the bioconversion process.

## Methods

**Fig. 10:**
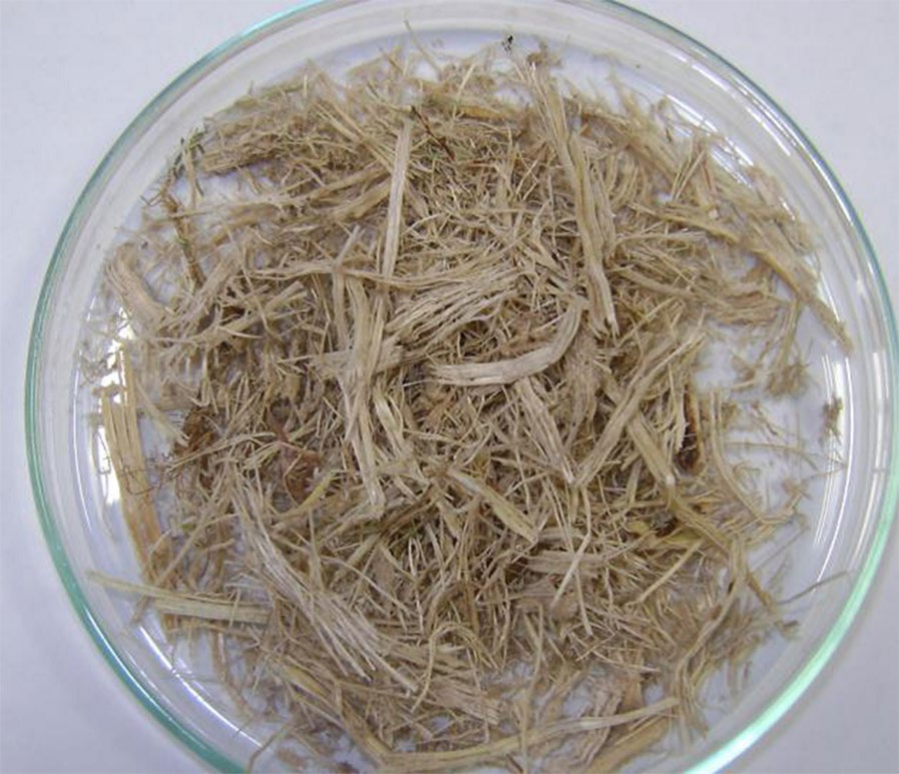
Sample of raw sugarcane bagasse

Fresh sugarcane bagasse was kindly provided by São Martinho sugar/ethanol plant (Pradópolis/SP-Brazil). It was dried at 60 °C to constant weight and kept into plastic bags in freezer. This biomass denominated raw bagasse was used as it comes from mill with different particle sizes (not passing through any step of screening), as shown in Fig. 10.

### Lime and hydrothermal pretreatments

Lime pretreatment was carried out as described by Chang, Nagwani, and Holtzapple [24]. In 500 mL flasks, raw bagasse (1 g dry weight) was treated with 100 mL of the calcium hydroxide solution (1 % w/v) in a ratio of 0.1 g lime per gram dry bagasse for 7, 30, and 60 min at 120 °C in an autoclave. For hydrothermal pretreatment, 100 mL of distilled water was added to raw bagasse (1 g dry weight) in 500 mL flasks and autoclaved under the conditions above. Subsequently, flasks were cooled at room temperature and the solid fraction (pretreated bagasse) was separated from hydrolysate by vacuum filtration, washed thoroughly with water to neutral pH and dried at 60 °C in an oven for 24 h. The dry weight obtained was used to determine pretreatment yield. All experiments were performed in duplicate.

### Compositional analysis

The chemical composition of untreated and pretreated raw sugarcane bagasse was determined according to analytical procedures established by NREL [25]. Raw bagasse samples (100 mg dry weight) were treated with 1 mL of sulfuric acid (72 % w/w) under vigorously stirring for 1 h at 30 °C. Thereafter, 84 mL of distilled water was added to the slurry and the mixture was kept at 120 °C for 1 h to complete oligosaccharides hydrolysis. After cooling, samples were filtered and the liquid phase was stored at −18 °C for subsequent analysis of total solids, ash, structural carbohydrates, and lignin.

The concentration of polymeric sugars (cellulose and hemicellulose) was determined from the concentration of the corresponding monomeric sugars, using an anhydro correction of 0.88 for C-5 sugars (xylose and arabinose) and a correction of 0.90 for C-6 sugars (glucose, galactose, and mannose).

The soluble lignin content present on liquid-phase samples was determined by measuring the absorbance at 240 nm on a UV–Visible spectrophotometer. For determination of insoluble lignin, the solid fraction was rinsed with water up to reaching neutral pH to remove acid residues and dried in oven at 105 °C until constant weight. Ash content was obtained by burning in muffle at 600 °C for 24 h. Total lignin was calculated as the sum of soluble and insoluble lignin fractions.

### Enzymatic hydrolysis

Enzymatic hydrolysis of pretreated raw sugarcane bagasse (insoluble fiber) was performed according to standard analytical procedures (LAP) described by NREL [26], using commercially available enzymatic preparation (Accellerase-1500^®^) kindly provided by Genencor International (Rochester, NY, USA). The enzymatic blend consisted of cellulase (15 FPU/g substrate) and β-glucosidase (75 U/g substrate) and the activities of these enzymes were determined according to methods described by Ghose [27].

Pretreated raw sugarcane bagasse samples were hydrolyzed in 50 mmol/L citrate buffer (pH 4.8) at a solid:liquid ratio of 1:100 (w/v) supplemented with enzymes and sodium azide (40 mg/L) to inhibit microbial contamination. This mixture was incubated at 50 °C for 72 h on a rotary shaker (150 rpm). All assays were performed in duplicate for each indicated time (12, 24, 48, and 72 h). Hydrolysate samples were collected, boiled to deactivate enzymes and analyzed for glucose and total reducing sugars.

The percentage of cellulose digestion was calculated by the ratio between the amount of cellulose digested and the amount of cellulose added, as shown in Eq. 1 [26].

1 $$ {\% }{\text{ Digestion}} = \frac{\text{Grams cellulose digested}}{\text{Grams cellulose added}} \times 100 $$

### Sugar measurements

Glucose and total reducing sugars were colorimetrically determined at 540 nm using GOD-PAP method [28] and DNS reagent [29], respectively. The xylose and arabinose concentration were colorimetrically measured at 671 nm using Bial’s reagent, according to method described by Pham et al. [30].

### Severity factor

The severity factor log(*R*_0_) was used to unify data obtained at different combinations of reaction time and pH of hydrothermal and lime pretreatments with respect to lignin solubilization. The factor *R*_0_, incorporating an integration of the time period used in the pretreatment done at a certain temperature, was calculated by Eq. 2:

2 $$ R_{0} = \int_{a}^{b} {\exp \left( {\frac{T(t) - 100}{14.75}} \right){\text{d}}t = t \times \exp } \left( {\frac{T(t) - 100}{14.75}} \right), $$

where *t* is the holding time of treatment in minutes, *T*(*t*) is the treatment temperature (in the time *t*), which 100 °C is the reference temperature [18]. The use of Eq (3) gives a more fair comparison of the pretreatment severities even at widely different pretreatment pH values [18].

3 $$ \log \left( {R_{0}^{{\prime \prime }} } \right) = \log \left( {R_{0} } \right) + \left| {{\text{pH}}\text{ - } 7} \right| $$

### Scanning electron microscopy (SEM)

The morphology of sugarcane bagasse before and after hydrothermal and lime pretreatments was examined by SEM. Samples were fixed with carbon tape on aluminum support (“stub”) and submitted to metal plating with 10 nm of gold in a sputter. Photomicrographs were obtained on Equipment TOP WITH SM 300 marks a power of electron beam 20 kV. Various images were obtained on different areas of samples in order to assure reliable results.

### Thermogravimetry (TG/DTA)

The thermogravimetric curves (TG) were obtained on a Netzsch termobalance, using alumina crucible, under the following conditions: heating rate of 10 °C min^−1^, temperature range 10–900 °C, oxidizing atmosphere with flow gas of 40 mL min^−1^. The derivative of TG curves (DTA) was obtained using TA analysis software.

### X-Ray diffraction (XRD)

Crystallinity of sugarcane bagasse before and after pretreatment was analyzed by X-ray diffraction in a Siemens D5000 Diffractometer employing Co-Kα radiation. Scans were obtained from 5° to 20° 2*θ* (Bragg angle) at a 0.05° per second of scanning rate. Powder sample data were recorded at room temperature.

The percentage of crystalline material in the biomass was expressed as the crystallinity index (I_c_), which was calculated by Eq. 4, following the procedure proposed by Segal et al. [31]:

4 $$ I_{\text{c}} = \frac{{\left( {I_{002} - I_{\text{am}} } \right)}}{{I_{002} }} \times 100 $$

in which *I*_002_ is the intensity of the 002 peak (2*θ* = 22º) and *I*_am_ is the intensity of the peak in the amorphous phase (2*θ* = 16º).

### Experimental design and data analysis

A full factorial design with repetition was applied to evaluate the main and interaction effects of time (7, 30, and 60 min) and type (lime or hydrothermal) of pretreatments on the raw sugarcane bagasse composition and saccharification performance.

The statistical significance of data was evaluated by analysis of variance (ANOVA) with confidence level of 95 %. The Minitab software version 15.0 (Minitab Inc., Pennsylvania) was used for the experimental design and for statistical analyses.

## Abbreviations

LAP: laboratory analytical procedures
NREL: national renewable energy laboratory
GOD-PAP: glucose oxidase/peroxidase-phenol-4-aminophenazone
DNS: dinitrosalicylic acid
FPU: filter paper units
U: enzyme unit
